# The Relationship between Cortisol and Bone Mineral Density in Competitive Male Cyclists

**DOI:** 10.1155/2013/896821

**Published:** 2013-05-29

**Authors:** Shannon L. Mathis, Richard S. Farley, Dana K. Fuller, Amy E. Jetton, Jennifer L. Caputo

**Affiliations:** ^1^Vanderbilt University Medical Center, Medical Center East-South Tower, Suite 4200, Nashville, TN 37232-8774, USA; ^2^Department of Health and Human Performance, Middle Tennessee State University, P.O. Box 96, Murfreesboro, TN 37132, USA; ^3^Department of Psychology, Middle Tennessee State University, P.O. Box 87, Murfreesboro, TN 37132, USA; ^4^Department of Biology, Middle Tennessee State University, P.O. Box 60, Murfreesboro, TN 37132, USA

## Abstract

*Objective*. The purpose of this study was to determine whether race day cortisol was related to bone mineral density (BMD) in competitive male cyclists. A secondary purpose was to determine additional factors associated with BMD in competitive male cyclists. *Methods*. Measurements of lumbar spine and hip BMD were performed in 35 male competitors in a state championship cycling time trial event. Salivary cortisol was measured 10 minutes prior to the start of the race and 5 minutes after race finished. Participants reported daily calcium intake, age, years of bike training, races per season, and average weekly minutes spent riding a bike, weight training, and running on a survey. *Results*. Cortisol level increased significantly from pre- to postcompetition but was not significantly associated with BMD. Increased weekly minutes of weight training was associated with higher BMD of the lumbar spine and the hip. The increased number of years of cycling experience was associated with lower BMD of the femoral neck. Increased daily calcium intake was associated with higher BMD of the lumbar spine and femoral neck. *Conclusions*. Findings indicate that cyclists should participate in weight training and increase calcium intake in order to increase or maintain BMD of the lumbar spine and hip.

## 1. Introduction

Interest in bone health among endurance athletes is growing. Studies showing low bone mineral density (BMD) in male cyclists are numerous [1–5], and osteopenia is prevalent in this population [4, 6]. Reports of lower BMD in competitive cyclists compared to age-matched controls are found in adult male cyclists [7], male master (over 30 years of age) cyclists [3], and in male postpubertal cyclists [8, 9]. Although not well documented, orthopedic injuries occur due to falls during cycling training and competition [10] making the occurrence of low BMD even more concerning. The etiology of low BMD in cyclists is multifactorial and may be partially due to an imbalance in hormones, such as cortisol [11, 12].

Cortisol triggers bone mineral resorption (removal) to free amino acids for use as an energy source through gluconeogenesis. Cortisol indirectly acts on bone by blocking calcium absorption which decreases bone cell growth [12]. The disruption to serum calcium homeostasis increases bone resorption [13] and ultimately reduces BMD [14]. Even a short bout of elevated cortisol secretion may cause a decrease in BMD [11, 12]. Excessive elevation of cortisol levels, such as in hypercortisolism or Cushing's syndrome, is linked to a high prevalence of osteoporosis and may be associated with the age-related decrease in BMD in the elderly [15].

Serum cortisol concentration increases due to physiological and psychological factors. A cyclist's resting levels of cortisol may increase in response to endurance training [16, 17], in response to intense cycling [18], and possibly in response to psychological arousal during competition [19–22]. Although the cortisol response specific to cycling competition has not been reported, researchers have reported elevations in cortisol on competition day due to anticipation of mental and physical performance [23]. Further, cortisol increased due to the physiological stress of competition as was indicated from pre- to postcompetition concentration [19]. 

The purpose of this study was to determine whether an elevation in cortisol before and/or after competition was related to BMD in male cyclists. Information garnered in this investigation determined whether cortisol levels at the start or finish of a competition were related to lumbar spine, total hip, femoral neck, or femoral trochanter BMD in competitive male cyclists. It was hypothesized that salivary cortisol would be correlated with BMD within the male cyclist study group. A secondary goal of the analysis was to investigate factors related to lumbar spine and hip BMD in competitive male cyclists.

## 2. Methodology

### 2.1. Participants

Middle Tennessee State University Institutional Review Board approval was gained prior to data collection. Amateur cyclists were recruited for participation in this study by contacting male competitors in a 40 km state championship time trial cycling competition on a course with rolling terrain. Among the participating cyclists (*N* = 35) were state, regional, and national competitors, with one former Olympian. Inclusion criteria consisted of cyclists over the age of 30 years, who trained consistently on a bicycle most days of the week and had at least 2 years of cycle-specific training experience. Exclusion criteria included use of medication known to affect the endocrine system and bone metabolism or a history of endocrine disorders. 

### 2.2. Instrumentation

#### 2.2.1. BMD Measurements

Lumbar spine (L1–L4), total hip, femoral neck, and trochanteric region areal BMD were measured using a Hologic Discovery QDR Series dual energy X-ray (DXA) absorptiometer (Bedford, MA, USA) either 1 week before or 1 week after the cycling competition. All scans were conducted and analyzed by a licensed technician using manufacturer protocols, and the DXA was calibrated prior to use. Adequate distributions were found to categorize cyclists as having either normal BMD (*T* > −1.0 SD) or low BMD (*T* ≤ −1.0 SD) according to the definitions by the World Health Organization (WHO) for use in statistical analyses [24, 25]. 

#### 2.2.2. Calcium Intake

Calcium intake was measured using a 1-day dietary recall performed during the week prior to the race on a day representative of the typical diet. Food intake, including vitamin supplements, was analyzed for daily calcium (mg/d) by the principle investigator using the US Department of Agriculture website (http://www.mypyramid.gov).

#### 2.2.3. Cortisol

Cortisol was measured 10 minutes prior to the start of the race and within 5 minutes of the finish. Cyclists were informed of the method of saliva collection prior to racing. The cyclists provided a saliva sample in a Salivette (Sarstedt, Newton, NC, USA), by placing a small cylindrical, nontoxic polymer oral swab under the tongue for 2 minutes. Saliva samples were stored on dry ice and were analyzed within 2 days of collection. Cortisol concentration within saliva was assayed in duplicate by radioimmunoassay (Hormone Assay and Analytic Services Core, Vanderbilt University, Nashville, TN, USA) with an intra-assay coefficient of variation of 3%. 

#### 2.2.4. Demographics

Participants reported age, number of years of bike-specific training, number of minutes spent lifting weights (min/week), and number of minutes spent running (min/week) on a survey. To ensure that participants consistently trained on a bicycle most days of the week, the average number of minutes spent riding a bike (min/week) and the approximate number of races per season were also reported.

#### 2.2.5. Prerace Nervousness

 Competitive state anxiety was used to measure prerace nervousness. Due to time sensitivity of gathering questionnaire data immediately prior to a competitive event, three items were chosen from the Competitive State Anxiety Inventory 2 [26]. A 4-point Likert-type scale was used to rate each item where a score of one represented “not at all” and a score of four represented “very much so.” Items for the shortened inventory were chosen to measure participants' cognitive and somatic anxiety. The items chosen were as follows: I am concerned about this competition; I feel nervous; and my body feels tense. An average of the score for the three items produced the final score for pre-race nervousness. The shortened inventory demonstrated good reliability (Cronbach's alpha = 0.70) in practice.

### 2.3. Procedures

All participants were informed of the procedures of the study and signed an informed consent document. Eligible participants reported to the University Exercise Science Lab either 1 week prior or 1 week after the state championship time trial competition. Upon arrival, cyclists completed the demographic questionnaire and the 1-day dietary recall. Cyclists were provided with hospital scrubs and removed shoes for all laboratory measurements. Measures of height and body mass were performed. Lastly, bone density scans were completed in randomized order. 

On the day of the race, cyclists reported to the start line where all measurements were performed 10 minutes prior to race start time. To avoid contamination of the saliva specimen from food or drink intake, cyclists were instructed to rinse their mouth with plain water and were not allowed to consume food or drink 10 minutes prior to saliva collection. A research assistant provided each cyclist with the competitive state anxiety questions on paper and a Salivette. As cyclists answered the questionnaire, the cotton swab from the Salivette was placed in the mouth for 2 minutes to allow full saturation. Immediately after race, the cyclists repeated the same procedure with a new Salivette. Saliva samples were placed on dry ice at the race site and transported to the laboratory for analysis.

### 2.4. Statistical Analyses

Anthropometric measurements, training characteristics, and cortisol concentrations before and after race are reported as mean ± standard deviation. Pearson Product Moment correlations were used to assess the relationship between cortisol (before and after race) and site-specific BMD for the study population. A two-way repeated measures ANOVA was conducted to determine whether cortisol levels (before and after race) were different for BMD categories defined as low (*T*-score ≤ −1.0) and normal (*T*-score > −1.0).

Linear regression analyses with stepwise selection were used to determine whether age, BMI, daily calcium intake, number of years cycling experience, number of races per season, number of minutes weight lifting (min/week), number of minutes running (min/week), prerace cortisol (nmol/L), postrace cortisol (nmol/L), and prerace nervousness were significantly associated with site-specific BMD. Due to the small sample size, three separate analyses were performed. The three variables for the first subgroup described participant demographics of age, BMI, and daily calcium intake. The second subgroup contained variables describing training regimen: years of cycling experience, number of races per season, weekly minutes of weight training, and weekly minutes of run training. Variables pertaining to hormonal and emotional characteristics, prerace cortisol, postrace cortisol, and prerace nervousness, were entered into the third stepwise linear regression. Significant variables from each subgroup were then combined into one final model to determine variables that were significantly associated with site-specific BMD. IBM SPSS version 19 was used to analyze the data. 

## 3. Results

Of the 35 participants, three cyclists were excluded due to a flat tire during the competition. Additionally, BMD measurements were not obtained in two cyclists, one prerace cortisol sample was contaminated, and eight postrace cortisol samples were contaminated. This resulted in 21 complete data sets. Demographic and BMD measurements were obtained in 30 participants. 

The cyclists' mean age was 42.1 years (SD = 9.0 years, CI = 38.8 years to 45.5 years). The cyclists average height was 180.4 cm (SD = 6.0 cm, CI = 178.2 cm to 182.6 cm), and weight was 80.9 kg (SD = 6.9 kg, CI = 78.3 kg to 83.5 kg). The cyclists participated in a mean of 22 (SD = 11, CI = 17 to 26) races per season and trained an average of 12.3 hr/wk (SD = 3.9 hr/wk, CI = 10.8 hr/wk to 13.8 hr/wk). The mean completion time for the 40 km time trial competition was 60 min (SD = 4 min, CI = 59 min to 62 min). Descriptive statistics for cyclists' demographics and training characteristics are shown in Table 1. Bone density measurements are shown in Table 2. Based on WHO classifications, 50% of the male cyclists had either lumbar spine or hip BMD classified as osteopenia and 40% of the cyclists had osteopenia of the lumbar spine. Correlations between pre- and postrace salivary cortisol and BMD of the lumbar spine, total hip, trochanter, and femoral neck are presented in Table 3.

### 3.1. Cortisol and Low versus Normal BMD

Means for pre- and postcompetition cortisol levels across BMD classifications of low BMD were 8.47 nmol/L (SD = 2.20) and 18.40 nmol/L (SD = 8.00), respectively, and normal BMD was 10.79 nmol/L (SD = 4.38) and 23.35 nmol/L (SD = 19.22), respectively. There was not a significant interaction between cortisol levels and BMD categories: *F*(1,19) = 0.14, MSE = 126.34, *P* = 0.71, and *ω* ^2^ = 0.00. There were no differences across BMD categories: *F*(1,19) = 1.25, *P* = 0.27, and *ω* ^2^ = 0.01. Cortisol level did increase significantly from pre- to postcompetition: *F*(1, 19) = 10.48, *P* = 0.004, and *ω* ^2^ = 0.31.

### 3.2. Variables Associated with BMD

#### 3.2.1. Associations with Lumbar Spine BMD

Of the participant characteristics, increased daily calcium intake was associated with higher lumbar spine BMD (*β* = 0.40, *t* = 2.30, and *P* = 0.029). Age and BMI were not associated with lumbar spine BMD in these athletes. Of the training characteristics, increased minutes of weekly weight training was associated with higher lumbar spine BMD (*β* = 0.61, *t* = 4.03, and *P* < 0.001). Measures of cycling experience, race frequency, and running (min/wk) were not associated positively or negatively with lumbar spine BMD. There were no significant associations between pre- or postrace cortisol or prerace nervousness and lumbar spine BMD, respectively. Daily calcium intake and weight training were entered in a single stepwise linear regression to determine associations with BMD, but only weight training was associated with lumbar spine BMD (*R* ^2^ = 0.37).

#### 3.2.2. Associations with Total Hip BMD

None of the participant characteristic variables (age, BMI, and daily calcium intake) were associated with total hip BMD. Of the training variables, increased minutes of weekly weight training was associated with higher total hip BMD (*β* = 0.62, *t* = 4.19, *P* < 0.001, and *R* ^2^ = 0.39). Measures of cycling experience and race frequency were not positively or negatively associated with total hip BMD nor did running (min/wk). There were no significant associations between pre- or postrace cortisol or prerace nervousness and BMD, respectively.

#### 3.2.3. Associations with Femoral Neck BMD

Of the participant characteristic variables, higher daily calcium intake was associated with higher femoral neck BMD (*β* = 0.37, *t* = 2.10, and *P* = 0.045). Age and BMI were not associated with femoral neck BMD in these athletes. Increased minutes of weight training per week was associated with higher femoral neck BMD (*β* = 0.72, *t* = 6.14, and *P* < 0.001), while increased years of cycling experience was associated with lower femoral neck BMD (*β* = −0.29, *t* = −2.60, and *P* = 0.015). Neither running (min/wk) nor competitive activity (races per season) was associated with femoral neck BMD. There were no significant associations between pre- or postrace cortisol or prerace nervousness and femoral neck BMD, respectively. Daily calcium intake, weight training, and years of cycling experience were entered in a stepwise regression analysis to determine associations with femoral neck BMD. Both weight training and years of cycling experience were associated with femoral neck BMD in the combined model (*R* ^2^ = 0.63). Results of this two-variable model for femoral neck BMD are presented in Table 4.

#### 3.2.4. Associations with Femoral Trochanter BMD

None of the participant characteristics (age, BMI, or daily calcium intake) were associated with femoral trochanter BMD. Increased weight training (min/wk) was associated with higher femoral trochanter BMD (*β* = 0.68, *t* = 4.92, *P* < 0.001, and *R* ^2^ = 0.46). Measures of cycling experience (years), competitiveness (races/season), and running (min/wk) were not associated with femoral trochanter BMD. There were no significant associations between prerace cortisol, postrace cortisol, or prerace nervousness and femoral trochanter BMD, respectively.

## 4. Discussion

The results of this study add to a growing body of literature pertaining to factors related to BMD in male, competitive cyclists. As indicated by *R* ^2^, an increase in weight training (min/wk) was associated with higher BMD of the lumbar spine (37%), total hip (38%), femoral neck (57%), and femoral trochanter (46%). Both weekly weight training and years of cycling experience together were used to create a statistical model that explained 63% of the observed changes in femoral neck BMD. There was a significant direct correlation (*r* = 0.62) between weight training and calcium intake; however, the reason for this correlation is unknown. As a result, post hoc analyses were performed to determine the contribution of calcium intake to each BMD site. Bivariate linear regression analyses showed that estimated daily calcium intake significantly explained 16% and 14% of the variance in lumbar spine and femoral neck BMD, respectively. The existing literature and present data have shown a prevalence of osteopenia in male cyclists [1, 2, 4, 6]. The present findings suggest that cyclists should include weekly weight training and consume adequate daily calcium to improve or maintain BMD. 

Raff et al. [15] measured cortisol and BMD in 130 men which is approximately four times the sample size of the present study. A significant negative correlation in a morning measurement of salivary cortisol and lumbar spine BMD was documented [15]. The nonsignificant correlation values in the present study may be due to the small sample size (*N* = 21). Cyclists typically train long hours with repeated bouts of moderate to high intensity work and participate in many competitive events eliciting repeated elevations of cortisol over a prolonged period of time [27, 28]. However, findings from the present study do not support the relationship between cortisol and BMD in cyclists and the notion that the decline in BMD may be partly due to excess glucocorticoid hormone secretion [4, 12]. 

Differences in cortisol concentration between cyclists with low BMD (*T* ≤ −1.0) and cyclists with normal BMD (*T* > −1.0) were not supported by these data. Cyclists with low BMD did not respond to a stressful event with a greater concentration of cortisol compared to cyclists with normal BMD. However, a significant increase in salivary cortisol concentration from pre- to postcompetition, where the time to complete the 40 km event ranged from 53 min to 67 min, was documented in the study. Under normal physiological conditions, salivary cortisol concentration would decline throughout the morning hours due to the circadian rhythm of the hypothalamic-pituitary-adrenal axis with a peak in the early morning hours and a nadir at night [28]. This finding suggests that a competitive cycling event provides strong psychological and physiological stimulation for the secretion of cortisol. This study also demonstrated that measurement of salivary cortisol offers a noninvasive alternative to serum as a method for assessing the hormonal response to competition stress in male cyclists [29]. 

Correlations between prerace nervousness and prerace cortisol were not supported in the present study. However, several studies have found significant correlations between preevent cortisol and measures of prerace nervousness [16, 18–23, 30]. Due to prerace time constraints in the present study, an abbreviated version of the Competitive State Anxiety Inventory 2 [26] was used. The use of the full scale may provide greater insight into the cycling competitor's prerace psychological state. 

Data relating cortisol to markers of bone mineral resorption are mixed. A disruption in the balance between bone mineral deposition and bone mineral resorption due to endurance cycling is hormonally mediated. Cycling induces secretion of parathyroid hormone (PTH) and type I collagen C (CTX), providing immediate measures of bone metabolism [31–33]. Researchers have suggested that bone loss in response to endurance cycling results from the mediation of cortisol with markers of bone resorption, possibly inducing an acceleration of bone resorption [4]. For example, CTX was positively associated with cortisol in cyclists during a laboratory cycling trial. Also, cortisol was once considered a regulator of PTH during exercise; however, data on this result are mixed [34].

Consistent with the existing literature, 40% of the cyclists in this sample had osteopenia of the lumbar spine and 50% of the cyclists had low BMD (*T* < 1.0) of the lumbar spine or hip [4, 6]. Participation in competitive cycling, where falling is prevalent, coupled with low BMD places cyclists at a high risk for fracture [3, 7]. Nichols et al. [3] recommended that cyclists have regular BMD screening, perform supplemental high-impact or weight training physical activity, and consume adequate calcium and vitamin D. The present findings support the recommendations of Nichols et al. [3]. Of the cyclists studied, 40% reported weekly participation in weight training (*M* = 28.7 min/wk, SD = 46.7 min/wk). Participation in weight lifting was positively associated with BMD of the lumbar spine, total hip, femoral neck, and femoral trochanter. It should be noted that 14 participants with low BMD reported participation in less than 20 min of weight lifting per week, whereas 10 participants with normal BMD reported participation in at least 30 minutes of weight lifting per week. In contrast to Nichols' et al. [3] suggestions, participation in running, a high-impact exercise, was not significantly associated with BMD in the present population.

Consistent with the recommendations by Nichols et al. [3], daily calcium intake was correlated with BMD of the lumbar spine and femoral neck. In addition, post hoc linear regression analyses revealed that daily calcium intake was significantly associated with higher lumbar spine and femoral neck BMD. Of the participating cyclists, 50% reported daily calcium intake greater than 1,200 mg/d. Evidence supports adequate intake of calcium and vitamin D to attenuate the loss of BMD in cyclists [3, 31].

Nichols and Rauh [35] measured BMD of master male cyclists in a 7-year longitudinal study. The cyclists experienced a greater decline in BMD compared to the nonathletes. Of the cyclists, 6 became osteoporotic during the 7-year period. However, participants that reported participation in weight training experienced less bone loss compared to those who did not. A meta-analysis by Specker [36] showed evidence from 17 trials that high-impact physical activity had a greater impact on BMD when high calcium intake was included. The author concluded that impact exercise and high calcium intake may not affect BMD independently, but impact activity may increase bone density when serum calcium is elevated.

Researchers have shown that dietary calcium intake does not influence BMD in cyclists [5, 7, 37]. According to Barry and Kohrt [37], there was no difference in BMD between cyclists taking a calcium supplement with meals and those without over the course of a year. However, researchers suggest that the timing of calcium supplementation is crucial to increasing BMD. Calcium intake before [33] or during [31] endurance cycling may provide a positive osteogenic effect. 

An association was found between the number of years of cycling experience and femoral neck BMD. An increase in the number of years of cycling experience was statistically associated with decreased femoral neck BMD. This confirms previous research on BMD of master cyclists. Nichols et al. [3] suggested that long-term participation in cycling may negatively impact BMD later in life. This assumption is presumably due to the accumulation of a high number of hours spent with supported body weight; a cyclist's body weight is supported by five contact points: two hands on the handlebar, two feet on the pedals, and the pelvis on the saddle. Pedal loads also relate strongly to the riders weight and body position during cycling, that is, seated or standing [38, 39]. 

This study provides original data on the assessment of pre- and postcompetition measurements of salivary cortisol with comparison to site-specific BMD in competitive male cyclists. Although the etiology of low BMD in male cyclists has yet to be determined, these data provide a clear and valuable contribution to the literature to determine the source of bone loss in competitive cyclists. Further research is needed to clearly delineate possible sources of bone loss in competitive and noncompetitive cyclists. Further, the high number of contaminated postrace salivary cortisol samples highlights the need for larger sample sizes in future research. In addition, testosterone, a hormone positively associated with markers of bone metabolism, may mediate bone resorption in concert with cortisol. For that reason, measuring the exercise-induced response of hormones such as prerace and postrace testosterone, PTH, and CTX may provide further insight into the hormonal mediation of accelerated bone resorption during intense cycling. 

In conclusion, salivary cortisol was not associated with BMD. On the basis of these data, BMD in male competitive cyclists was not related to the increase in cortisol due to the physiological or psychological stress of competition. However, findings support the relationship between weekly participation in weight training and higher BMD of the lumbar spine and hip. The findings also support a positive relationship between daily calcium intake and BMD of the lumbar spine and femoral neck. It is recommended that cyclists participate in weight training and increase daily calcium intake in order to increase or maintain BMD.

## Figures and Tables

**Table 1:** Means, standard deviations, and 95% confidence intervals for cyclist characteristics.

Variable	M	SD	95% CI
Age	42.1	9.0	[38.8, 45.5]
BMI	24.8	1.5	[24.3, 25.4]
Calcium intake	1313.9	547.0	[1109.7, 1518.2]
Years of cycling experience	11.7	9.7	[8.1, 15.4]
Races per season	21.5	11.3	[17.3, 26.7]
Bicycle training	12.3	3.9	[10.8, 13.8]
Weight training	28.7	46.7	[11.2, 46.1]
Run training	13.2	40.2	[−1.7, 28.3]
Race completion time	60.8	3.9	[59.2, 62.4]
Prerace cortisol^a^	9.4	4.1	[7.8, 11.0]
Postrace cortisol^b^	20.8	14.5	[14.3, 27.2]
Prerace nervous	2.6	0.7	[2.3, 2.9]

Note. ^a^ *n* = 29, ^b^ *n* = 22; BMI represents body mass index. Calcium intake was estimated as mg/day. Bicycle training was measured in hr/wk. Weight training and run training were measured in min/wk. Race completion time was measured in min. Salivary cortisol was measured in nmol/L.

**Table 2:** Means, standard deviations, and 95% confidence intervals for BMD (*N* = 30).

Variable	M	SD	95% CI
g/cm^2^			
Lumbar spine	1.033	0.12	[0.988, 1.077]
Total hip	0.971	0.12	[0.928, 1.015]
Femoral neck	0.817	0.12	[0.772, 0.860]
Femoral trochanter	0.738	0.10	[0.700, 0.861]
*T*-scores			
Lumbar spine	−0.51	1.08	[−0.92, −0.11 ]
Total hip	−0.35	0.77	[−0.64, −0.06]
Femoral neck	−0.81	0.87	[−1.14, −0.49]
Femoral trochanter	−0.30	0.78	[−0.59, −0.003]

**Table 3:** Correlations for BMD and cyclist characteristics (*N* = 30).

Variable	1	2	3	4	5	6	7	8	9	10	11	12	13
Predictor variable													
(1) Age	—												
(2) BMI	0.00	—											
(3) Calcium intake	0.07	0.07	—										
(4) Weight training	0.01	0.31	0.62^†^	—									
(5) Years of cycling	0.67^†^	−0.05	0.00	−0.12	—								
(6) Run training	−0.14	0.42∗	−0.16	0.20	−0.23	—							
(7) Races per year	−0.11	−0.26	0.28	−0.19	0.24	−0.42∗	—						
(8) Prerace cortisol^a^	−0.05	0.31	0.15	0.25	0.08	0.29	−0.10	—					
(9) Postrace cortisol^b^	−0.02	−0.24	0.00	−0.23	−0.11	0.19	−0.02	−0.08	—				
(10) Prerace nervousness	−0.16	−0.27	0.06	−0.03	−0.05	−0.21	0.05	−0.19	−0.08	—			
Dependent variables													
(11) Lumbar spine	0.02	−0.11	0.40∗	0.61^†^	−0.22	−0.44	−0.22	−0.04	0.03	0.03	—		
(12) Total hip	−0.24	0.16	0.21	0.66^†^	−0.28	0.13	−0.28	0.16	−0.02	−0.03	0.76^†^	—	
(13) Femoral neck	−0.27	0.08	0.38∗	0.75^†^	−0.37∗	0.12	−0.28	0.11	−0.08	−0.11	0.86^†^	0.91^†^	—
(14) Femoral trochanter	−0.01	0.05	0.29	0.64^†^	−0.17	0.09	−0.24	0.14	−0.01	0.10	0.82^†^	0.90^†^	0.86^†^

Note*.* ^a^ *n* = 29, ^b^ *n* = 22; ^†^denotes that correlation is significant at the 0.01 level; ^*^denotes that correlation is significant at the 0.05 level. BMI represents body mass index. Calcium intake was estimated as mg/day. Weight training and run training were measured in min/wk. Salivary cortisol was measured in nmol/L. BMD was measured as g/cm^2^.

**Table 4:** Linear regression analysis summary for cyclist characteristics and femoral neck BMD (*N* = 30).

Variable	*B*	SE *B*	*β*	*t*	*P*
Weight training (min/wk)	0.01	0.002	0.72	6.39	<0.001
Years of cycling experience	−0.03	0.010	−0.29	−2.60	0.015

Note. Variable contribution to femoral neck BMD: weight training, *R* ^2^ = 0.57; years of cycling experience *R* ^2^ = 0.14.
